# Supplementary material for: Genetic Algorithm Design of MOF-based Gas Sensor Arrays for CO2-in-Air Sensing
Source: Sensors (Basel). 2020 Feb 10;20(3):924. doi: 10.3390/s20030924 (PMC7039381; doi:10.3390/s20030924)
Supplement: Supplementary file 1 [file sensors-20-00924-s001.pdf]

Genetic Algorithm Design of MOF-based Gas Sensor Arrays for CO_2-in-Air Sensing

Brian A. Day [1] **and Christopher E. Wilmer** [1,2,*]

[1] Department of Chemical and Petroleum Engineering, University of Pittsburgh, 3700 O'Hara St, Pittsburgh, PA 15261, USA; brd84@pitt.edu
[2] Department of Electrical and Computer Engineering, University of Pittsburgh, 3700 O'Hara St, Pittsburgh, PA 15261, USA
* Correspondence: wilmer@pitt.edu

Supplementary Information

Table of Contents

1. RASPA Simulation Details
2. Simulating Sensor Measurements
   2.1. Element Probabiity Values
   2.2. Array Probability Values
   2.3. Component-wise Probability
   2.4. Kullbeck-Leibler Divergence
3. Screening Arrays
   3.1. Brute Force Analysis
   3.2. Genetic Algorithm Analysis
4. References

---

1. *RASPA Simulation Details: Forcefield, LJ Params, MOF cif Files Source, Initialization/Run Cycles*

Adsroption data was generated using RASPA, a grand canonical Monte Carlo simulation software designed by Duddledam et al. [1] To model CO_2 in air, we studied a set of ternary gas mixtures containing CO_2, O2, and N_2. The compositions of CO_2 and O_2 ranged from 0% to 30%, and the composition of N_2 ranged from 40% to 100%, each in increments of 1%. This resulted in 961 unique gas mixtures. We then ran GCMC simulations to calculate the adsorption of each gas mixture in a set of 50 MOFs from the CoRe MOF database [2] at a temperature of 298 K and a pressure of 1 bar, reflecting ambient conditions. Simulations were conducted using 1000 initialization cycles and 2000 priduction cycles. A single cycle consists of n Monte Carlo steps, where n is equivalent to the number of molecules in the simulation. Note that this value fluctuates during a GCMC simulation. The simulations include the following moves: insertion, deletion, translation, regrowth (configuration is changed), and swapping.

To model electrostatic interactions, which are important for accurately predicting CO_2 and, to a lesser extent, N_2 adsorption, we assigned partial charges to the atoms of the MOF frameworks via the EQeq method [3]. Similarly, the molecule parameters of the gases also included partial charges, and the forcefield which we used, TrAPPE [4], has been shown to accurately simulate these effects.

Rigid MOF structures, as well as rigid molecule strucutures, were assumed, and Lennard-Jones (LJ) potentials with a cutoff of 12 Å were used along with Ewald charge interactions to determine the overall energy of the structure and adsorbed gases. The equations for LJ potential is are given below, where ε is potential well-depth and σ is radius of interaction.

$$V_{ij} = 4\varepsilon_{ij}\left[\left(\frac{\sigma_{ij}}{r_{ij}}\right)^{12} - \left(\frac{\sigma_{ij}}{r_{ij}}\right)^{6}\right]$$

The equation for Ewald coulombic potential in a periodic system is given as:

$$U^{sys} = U^{real} + U^{rec}$$

$$U^{real} = \sum_{i<j} q_i q_j \frac{erfc(\alpha r_{ij})}{r_{ij}}$$

$$U^{rec} = \frac{2\pi}{V}\sum_{k}\frac{1}{k^2}e^{-\frac{k^2}{4\alpha^2}}\left(\left|\sum_{i=1}^{N} q_i \cos(\boldsymbol{k}\cdot\boldsymbol{r}_i)\right|^2 + \left|\sum_{i=1}^{N} q_i \sin(\boldsymbol{k}\cdot\boldsymbol{r}_i)\right|^2\right) - \sum_{i}\frac{\alpha}{\sqrt{\pi}}q_i^2$$

where q_i and q_j are the charges of particle i and j, respectively, $\boldsymbol{r_i}$ is the position of atom i, V is the volume of the cell, α is a damping factor, k is the wavelength, and erfc is the error function complement.

The information about each framework, including minimum number of unit cells, density, volumetric surface area, void fraction, and pore size (largest cavity diameter) are listed below in Table S1. Forcefield parameters (excluding partial charges, which are framework specific and can be found in the cif files) for each framework atom are given in Table S2.

Table S1. Physical Properties of MOF Structures

MOF	Unit Cells [a, b, c]	Density [g/cm^3]	Surface Area [m^2/cm^3]	Void Fraction [---]	Pore Size [Å]
IRMOF-1	1, 1, 1	0.590375	2198.21	0.8108	15.08377
HKUST-1	1, 1, 1	0.879099	2114.54	0.7206	13.18983
NU-125	1, 1, 1	0.57834	2196.18	0.79	19.37323
UIO-66	2, 2, 2	1.22494	1762.62	0.6128	8.88
ZIF-8	2, 2, 2	0.924676	1442.14	0.6416	11.51766
MgMOF-74	1, 1, 4	0.91487	1549.21	0.6396	11.63962
MOF-177	1, 1, 1	0.426775	2035.73	0.8318	11.67849
NU-100	2, 2, 2	0.2843005	1620.675	0.8777	27.190265
MOF-801	1, 1. 1	1.74184	1303.21	0.5322	7.65165
ALUKIC	2, 2, 1	0.56692	2883.21	0.7934	8.54387
AMIMAL	2, 1, 1	0.988926	1269.07	0.6132	11.07211
AXUHEH	2, 2, 1	1.06453	1024.66	0.4958	7.21454
BAZGAM	1, 1, 1	0.126526	810.47	0.9392	42.79818
BIWSEG	1, 1, 1	0.466941	1434.98	0.843	29.73511
EDUVOO	2, 2, 2	0.373403	1788.47	0.862	20.93415
FIDRIV	2, 2, 2	0.698397	1517.46	0.7102	15.99327
GAGZEV	1, 1, 1	0.279149	1594.77	0.8816	28.66522
GUPBEZ	2, 2, 2	2.5399	489.614	0.4518	7.29165
HABQUY	1, 1, 1	0.289452	1646.58	0.8738	25.71531
HIFTOG	2, 2, 2	1.16582	1897.92	0.6126	7.95891
JEWCAP	2, 2. 1	1.11472	880.16	0.5446	6.52252
KICXAX	2, 2, 2	3.58491	368.423	0.3658	5.16268
KIFJUF	3, 2. 2	0.821526	2412.21	0.648	5.86033
KINKAV	3, 2, 2	1.21016	462.625	0.4526	4.49986
LODPUQ	2, 2, 2	1.07351	1496.3	0.5402	6.04771
LOFVUY	2, 1, 1	1.07811	1889.15	0.626	8.04365
MUDTEL	1, 1, 1	0.559282	2154.91	0.789	19.09514

NAYZOE	2, 2, 2	0.498918	2302.42	0.813	15.82314
NIBHOW	1, 1, 1	0.279595	1425.71	0.8844	27.51057
NIBJAK	1, 1, 1	0.223433	1188.94	0.9102	32.00355
OFEREX	3, 3, 2	1.56791	1544.85	0.5694	6.99117
RAVXET	4, 1, 1	0.326773	945.437	0.8304	38.22812
RAVXIX	4, 1, 1	0.23463	734.576	0.8668	53.57674
RAVXOD	4, 1, 1	0.179103	619.991	0.8986	71.64119
RUTNOK	1, 1, 1	0.240823	1468.58	0.9018	24.61263
SADLEQ	3, 3, 2	1.50504	1508.7	0.5702	7.11187
SAPBIW	1, 1, 1	0.305675	915.118	0.889	28.19349
SICZOV	2, 2, 2	0.419881	1773.06	0.8408	18.76086
TOHSAL	2, 2, 1	0.576207	2737.54	0.7668	9.79069
UKUPUL	2, 2, 2	1.43379	1465.65	0.5222	6.90717
VETTIZ	1, 1, 1	0.537597	1117.38	0.7638	21.62456
WIYMOG	2, 2, 1	0.408102	2874.44	0.8306	12.0545
WUNSEE01	2, 2, 2	1.20903	749.985	0.4842	5.00556
XAFFAN	2, 2, 2	0.365184	1896.05	0.8544	14.91316
XAFXOT	6, 3, 2	1.88819	647.785	0.3646	5.91185
XAHQAA	1, 1, 1	0.170429	1040.62	0.9292	23.03533
XALTIP	2, 2, 2	0.551216	1809.24	0.7988	18.68299
XUKYEI	2, 2, 2	0.287208	1805.38	0.8682	13.17229
XUWVUG	7, 2, 2	3.19434	197.336	0.287	3.95338
YEQRIV	3, 2, 2	0.74227	3172.25	0.734	5.99633

Table S2. Parameters of Framework Atoms

Atom Type	ε/k_B [K]	σ [Å]	Atom Type	ε/k_B [K]	σ [Å]
H	22.1417	2.886	Co	7.04507	2.55866
Be	42.7736	2.44552	Ni	7.54829	2.52481
B	47.8058	3.58141	Cu	2.5161	3.495
C	52.8381	3.851	Zn	62.3992	2.46155
N	34.7222	3.66	Ga	208.836	3.90481
O	30.1932	3.5	As	155.47	3.77
F	36.4834	3.092	Br	186.191	3.51905
Na	15.09	2.66	Zr	34.7221	3.124
Mg	55.8574	2.69141	Ag	18.1159	2.80455
Al	155.998	3.91105	Cd	114.734	2.53728
Si	155.998	3.80414	In	301.428	3.97608
P	161.03	3.69723	Sb	225.946	3.93777
S	173.107	3.59032	Te	200.281	3.98232
Cl	142.562	3.51932	I	170.57	4.01
K	17.61	3.4	La	8.55	3.14
Sc	9.56117	2.93551	Ce	6.54	3.17
Ti	8.55473	2.8286	Nd	5.03	3.18
V	8.05151	2.80099	Eu	4.03	3.11
Cr	7.54829	2.69319	Tb	3.52	3.07
Mn	6.54185	2.63795	Dy	3.52	3.05
Fe	6.54185	2.5943	W	33.71	2.73

Table S3. Parameters of Gas Molecule Bodies

	Atom Type	X [Å]	Y [Å]	Z [Å]	ε/k_B [K]	σ [Å]	Charge [e]
**(0)**	O_CO_2	0.0	0.0	1.16	79.0	3.05	-0.35
**(1)**	C_CO_2	0.0	0.0	0.0	27.0	2.80	0.70
**(2)**	O_CO_2	0.0	0.0	-1.16	79.0	3.05	-0.35
**(0)**	N_N_2	0.0	0.0	0.55	36.0	3.31	-0.482
**(1)**	N_COM*	0.0	0.0	0.0	---	---	0.964
**(2)**	N_N_2	0.0	0.0	-0.55	36.0	3.31	-0.482
**(0)**	O_O_2	0.0	0.0	0.605	49.000	3.02	-0.113
**(1)**	O_COM*	0.0	0.0	0.0	---	---	0.226
**(2)**	O_O_2	0.0	0.0	-0.605	49.000	3.02	-0.113

*COM = Center of Mass

The Peng-Robinson equation of state, shown below, was used to calculate the fugacities necessary to run the GCMC simulation. The critical parameters for each molecule type are listed below in table S4.

$$p = \frac{RT}{V_m - b} - \frac{a\alpha}{V_m^2 + 2bV_m - b^2} \text{ where } a = \frac{0.457235R^2T_c^2}{p_c} \;\&\; b = \frac{0.077796RT_c}{p_c}$$

$$\alpha = \left(1 + k(1 - T_r^{0.5})\right) \text{ where } k = 0.37464 + 1.54226\omega - 0.26992\omega^2 \;\&\; T_r = T/T_c$$

Table S4. Critical Parameters of Gas Molecules

Molecule Type	T_C [K]	P_C [MPa]	ω	Bond Stretch
CO_2	304.1282	7.377300	0.22394	Rigid
N_2	126.192	3.395800	0.0372	Rigid
O_2	154.581	5.043000	0.0222	Rigid

2. *Simulating Sensor Measurements*

2.1. Element Probability Values

The following section is intended to give an overview of the of the calculations involved in designing arrays and predicting compositions. Specific information about formatting the results and controlling certain parameters of the code is available on the GitHub which hosts this project (https://github.com/WilmerLab/sensor_array_mof_adsorption). This work is a continuation of previous work done by Gustafson et al. [5–7]

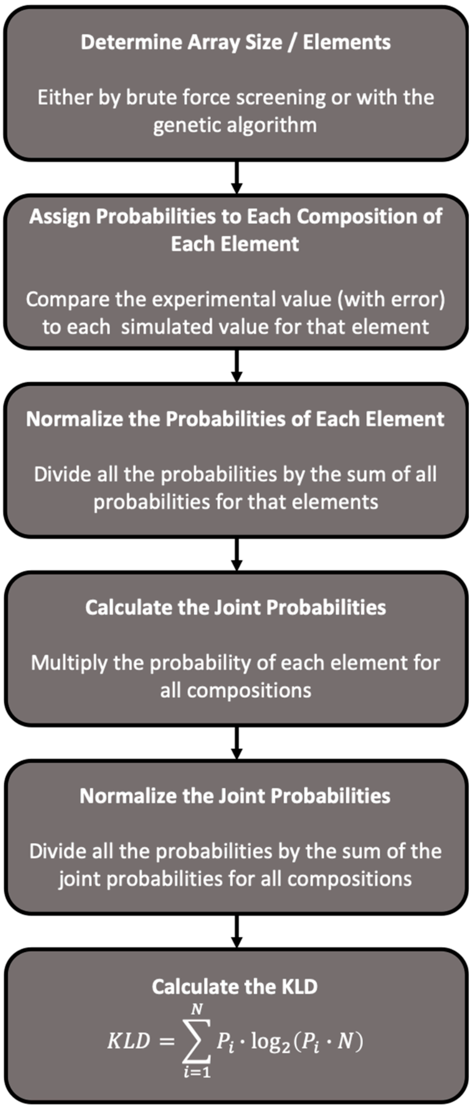

Figure S1. Overview of Analysis Procedure

For the work presented in this paper, we first needed to perform calculations for gas mixtures of CO_2, O2, and N_2. The compositions of CO_2 and O2 ranged from 0% to 30%, and the composition of N_2 ranged from 40% to 100%, all in increments of 1%, yielding a total of 961 gas mixtures, and for 50 MOFs, a total of 40,850 distinct simulations. Once these calculations were complete, we would accumulate all of the adsorbed mass values, and create two distinct sets of data; the simulated masses, which included the adsorbed mass values for all MOFs and all compositions, and the experimental mass values, which is a subset of the simulated mass values and includes data for all MOFs, but for only a sinlge composition. With these two data sets, we could begin the analysis.

After loading the data, the first step is to calculate the probability of each composition for each MOF. One MOF at a time, we take the experimental value associated with that MOF and create a truncated normal probability curve centered about the experimental mass, with a standard deviation 5% of the experimental mass. The intention of using a truncated probability distribution rather than a true normal distribution is to account for the fact that

adsorption will always result in an increase in mass. Consequently, the lower bound is set at 0, and the upper bound is set far beyond the highest simulated mass present in the data set.

The equations which govern the truncated normal distribution are as follows:

$$\psi(\bar{\mu},\bar{\sigma},a,b;x) = \begin{cases} 0 & if\ x \le a \\ \dfrac{\phi(\bar{\mu},\bar{\sigma}^2;x)}{\Phi(\bar{\mu},\bar{\sigma}^2;b) - \Phi(\bar{\mu},\bar{\sigma}^2;a)} & if\ a < x < b \\ 0 & if\ b \le x \end{cases}$$

$$\phi(\bar{\mu},\bar{\sigma}^2;x) = \frac{1}{\sigma\sqrt{2\pi}} e^{-\frac{(x-\mu)^2}{2\sigma^2}}$$

$$\Phi(\bar{\mu},\bar{\sigma}^2;x) = \int_{-\infty}^{x} \frac{1}{\sigma\sqrt{2\pi}} e^{-\frac{(t-\mu)^2}{2\sigma^2}} dt$$

where $\phi(\bar{\mu},\bar{\sigma}^2)$ is the standard normal distribution over the interval $(-\infty,+\infty)$, and $\Phi(\bar{\mu},\bar{\sigma}^2)$ is the cumulative distribution function over the interval $(-\infty,+\infty)$. The variables $\bar{\mu}$ and $\bar{\sigma}$ is the mean and variance of the parent normal distribution, and the variables a and b are the truncation interval [8].

For each composition, we assign a probability based on where simulated mass sits on the truncated probability curve, as given by:

$$P_{sim,i} = \psi\left(\bar{\mu},\bar{\sigma},a,b;m_{sim,i}\right)$$

where $\bar{\mu} = m_{exp}$, $\bar{\sigma} = 0.05 \cdot m_{exp}$, $a = 0$, and $b = 1.05 \cdot m_{sim,max}$. Since each mass is assigned a probability independently of each other, the sum of all probabilities does not necessarily equal 1. However, since the intention of this process is to determine which simulated composition we have exposed the array to, we normalize the assigned probabilities so that now their sum equals 1.

$$F = \sum_{i=1}^{N} P_{sim,i}$$

$$P_{sim,i}^{norm} = \frac{1}{F} \cdot P_{sim,i}$$

This process is repeated for each MOF until we have one normalized probability value for each composition for each MOF.

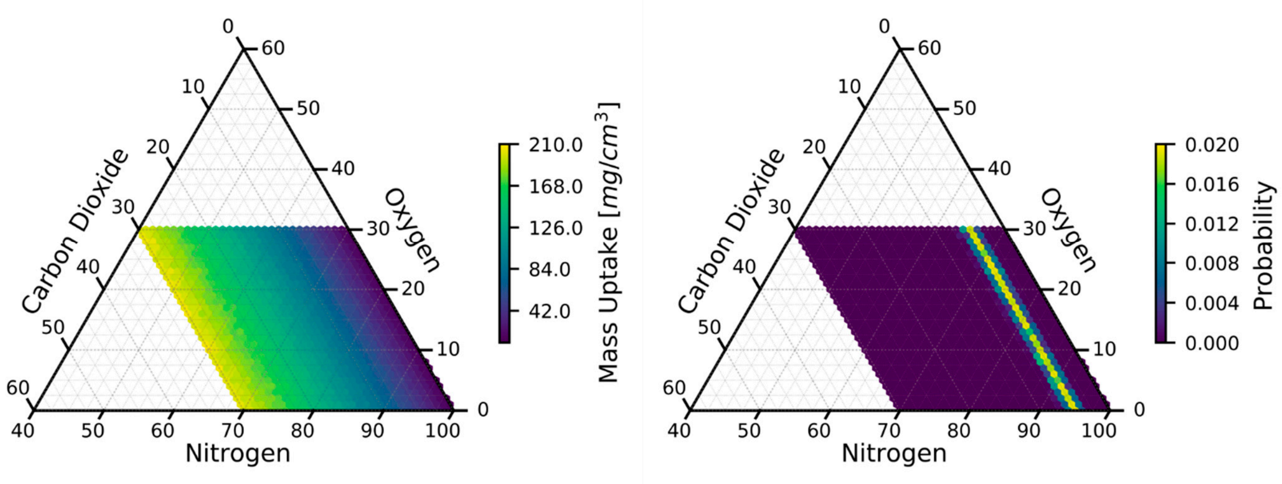

Figure S2. Mapping of Mass uptake to probability as a 1-element sensor of Mg-MOF-74, the top performing 1-element sensor.

2.2. Array Probability Values

Now that we have the probabilities for each MOF, we need to determine the probabilities for arrays. Fortunately, this process is very straightforward. For each composition, we simply multiply all of the normalized probabilities for each MOF with each other, resulting in a non-normalized array probability for each composition. As before, we normalize these probabilities so that they sum to 1. With this information, we can now say to which of the simulated gas mixtures the array has most likely been exposed. Figure S2 shows a mapping of mass values to probabilities for a composition corresponding to 5% CO_2, 20% N_2, and 75% O_2.

2.3. Component-wise Probability

In addition to calculating the probability for each of the simulated compositions, it is often convenient to be able to predict the mole fraction of each component individually. To this end, we developed a simple approach leveraging the previously calculated probabilities. For whichever component we are trying to predict, we establish a set of bins, typically with the same spacing as the simulated compositions, though this stipulation is not required. Then, for each of the simulated compositions, we assign it to its corresponding component bin. For example, if our bin boundaries for a given CO_2 bin were 29.5 and 30.5, all compositions where the mole fraction

of CO_2 was 30% would be placed in that bin. Next, we sum all of the probabilities in that bin to determine the total probability for that bin. Note that since this method uses the already normalized probabilities, the sum of the probabilities for each bin already equals one, and thus no additional normalization is needed.

We can repeat this process for each component in the mixture, until the mole fraction for each component has been predicted individually, though this is not necessary, and may often be undesirable. Nevertheless, it is important to note that if we were to use this approach to individually predict the mole fraction of all components, we are not guaranteed to predict the same composition as we had predicted the when considering the mixture as a whole. Furthermore, the sum of each of the mole fractions is not guaranteed to equal one, however both of these scenarios become less likely as the quality of the array improves.

It seems worthwhile to mention the advantages of this approach, as the previously mentioned scenarios would seem to demotivate using it. Notably, one could conceive developing an array which is uniquely sensitive to one primary component (or a set of primary components), and less sensitive to the remaining gases of a typical mixture. It is then conceivable that the array would continue to predict the primary (set of) component(s) reliably, regardless of how the mole fractions of the remaining components fluctuate. It is additionally possible that the array would continue predicting reliably in the presence of other gases which were not accounted for in the simulations. Conversely, by trying to predict the mixture as a whole, it is foreseeable that in either of these cases, the prediction of the component(s) of interested in negatively impacted by the less important components. Although none of the above situations are guaranteed to hold for all mixtures or arrays, hopefully they demonstrate at least the advantage of having this method available.

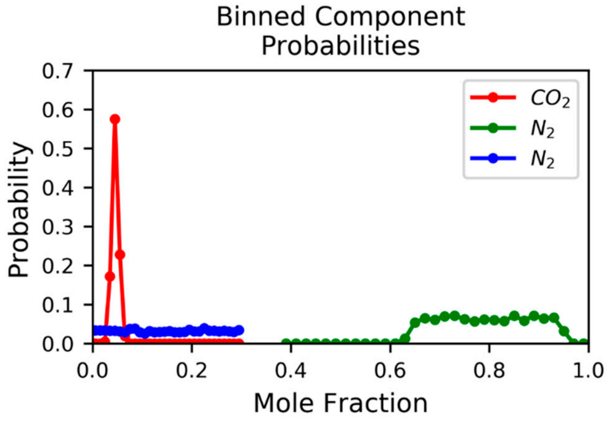

Figure S3. Component-wise probability for MgMOF-74, the top-performing single-element sensor.

2.4. Kullbeck-Liebler Divergence (KLD)

Although the set of probabilities for an array enables us to predict the composition of the mixture, it does not lend itself conveniently to comparing the quality of different arrays. Consequently, we wanted a way of quantifying the prediction capabilities of an array, and for this purpose we introduced the Kullbeck-Liebler divergence (KLD).

Rigorously, the KLD [9] quantifies the difference between two probabilities of any form and can be represented mathematically as follows:

$$KLD(P||Q) = \sum_{i=1}^{N} P_i \cdot \log_2\left(\frac{P_i}{Q_i}\right)$$

where P and Q are the system and reference probability respectively. When the reference probability is simply a uniform distribution (i.e. random chance), $Q_i = \frac{1}{N}$ for all I, so this simplifies to:

$$KLD = \sum_{i=1}^{N} P_i \cdot \log_2(P_i \cdot N)$$

Note that we also drop the $(P||Q)$ notation, since our reference probability is never anything other than a uniform distribution. This form of the equation can be used both when trying to predict the mixture as a whole and when trying to predict the mixture component-wise, the only difference is that the number of points, N, changes. We have taken to calling the these the

absolute KLD and component KLD respectively. We also calculate what we have been calling the joint KLD, which is simply the product of all of the component KLDs, though there does not seem to be any advantage to using this in place of the absolute KLD.

3. *Screening Arrays*

3.1. Brute Force Array Analysis

In order to determine all possible arrays of a given size, we simply iterate overall available MOFs, repeating this up to the number of elements in the array, and add a MOF only when it has not previously appeared in the array. Once all arrays have been determined, the compound probability, and subsequently the KLD, is evaluated as described above. We can then rank all arrays on the basis of KLD (or component KLD, or any other numerical property of interest), to find the best and/or worst arrays.

3.2. Genetic Algorithm – Explain mutation strategy

With 50 MOFs to choose from, there are over $2.1\text{x}10^6$ possible 5-element arrays. With an array size of 25 elements, there are over $1.25\text{x}10^{14}$ possible arrays, thus motivating the need for an intelligent screening approach to study these larger arrays. The number of possible arrays for a given array size can be calculated as:

$$\binom{N}{M} = \frac{N!}{M!\,(N-M)!}$$

where N is the number of possible MOFs, and M is the number of MOFs in an array. To this end, we developed a genetic algorithm which works in the following way.

Before explaining the details of our genetic algorithm approach, let us first cover some basic terminology. The ‘genetic’ in genetic algorithm refers to the fact that we are using distinct pieces of information about an array to modify it. Here the ‘genes’ correspond to the individual elements in the array. A generation refers to a distinct set of arrays, with a subset of each generation, the parents, being used in creating the following one. In our particular approach, all of the selected parents are also part of the next generation which they are used to create. This strategy, known as elitism, guarantees that quality of the solution does not decrease between

generations. Finally, the individual arrays of the following generation, created from the parent arrays, are known as children.

To begin the algorithmic search, an initial generation of arrays is first created at random (checking to make sure there are no duplicate elements in a single array, and no duplicate arrays in a single generation). Once created, their compound probability, and subsequently KLD, is evaluated as described above. The arrays are then ranked based on the property of interest, typically the KLD or one of the component-KLDs.

In order to create the next generation of arrays, we take a fixed number of the top performing arrays (or bottom two, if seeking the worst performing arrays), along with a fixed number of the remaining arrays at random. These arrays are both part of and parents for the next generation.

There are two approaches which we can use in creating children; crossover and mutation. With crossover, we choose two parents and generate a child from the elements contained in each. With mutation, as seen in Figure S4, we choose a single parent and go through each element one at a time. For each element, we generate a random number between 0 and 1. If the number we generate is less than our chosen mutation rate (another number between 0 and 1), than we replace that element with one of the MOFs not currently in the array. If the number we generate is greater than the mutation rate, the element remains in the array. Either one or both of these strategies can be used in creating children for the next generation, however we found that mutation strategies worked best for this application, and thus all of the results presented in the paper use only mutation.

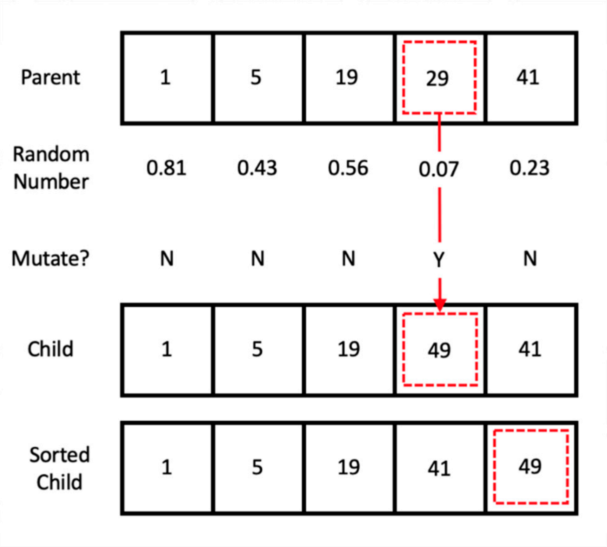

Figure S4. Example decision process for the mutation strategy employed in the genetic algorithm.

This entire process is repeated for the desired number of generations, and typically the genetic algorithm is run multiple times. For the results presented in this paper, the parameters were as follows: 20 arrays per generation, top 2 arrays were used as parents, along with 2 at random, and 200 generations per run. We used a variable mutation rate throughout the process. For the first 25 generations, the mutation rate was 50%, for the next 25 generations it was 25%, the next 50 generations used 10%, followed by another 50 generation at 5%, and lastly 50 generations at 2%. For each array size, the genetic algorithm was run no less than 3 times for seeking both the best and worst arrays, for a minimum of 6 runs.

A flowchart overviewing this process is given below in Figure S5:

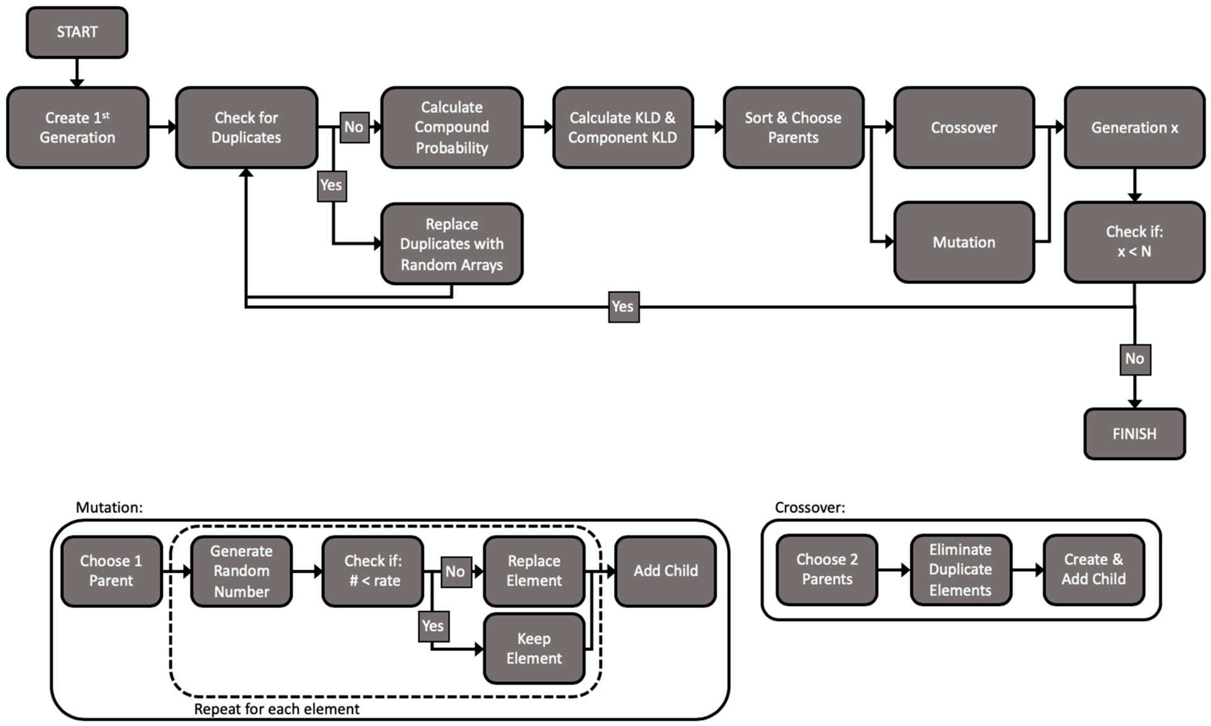

Figure S5. Flowchart overview of the genetic algorithm.

4. References

1. Dubbeldam, D.; Calero, S.; Ellis, D.E.; Snurr, R.Q. RASPA: molecular simulation software for adsorption and diffusion in flexible nanoporous materials. *Mol. Simul.* **2016**, *42*, 81–101. https://doi.org/10.1080/08927022.2015.1010082.
2. Chung, Y.G.; Camp, J.; Haranczyk, M.; Sikora, B.J.; Bury, W.; Krungleviciute, V.; Yildirim, T.; Farha, O.K.; Sholl, D.S.; Snurr, R.Q. Computation-Ready, Experimental Metal–Organic Frameworks: A Tool To Enable High-Throughput Screening of Nanoporous Crystals. *Chem. Mater.* **2014**, *26*, 6185–6192. https://doi.org/10.1021/cm502594j.
3. Wilmer, C.E.; Kim, K.C.; Snurr, R.Q. An Extended Charge Equilibration Method. *J. Phys. Chem. Lett.* **2012**, *3*, 2506–2511. https://doi.org/10.1021/jz3008485.
4. Martin, M.G.; Siepmann, J.I. Transferable Potentials for Phase Equilibria. 1. United-Atom Description of n-Alkanes. *J. Phys. Chem. B* **1998**, *102*, 2569–2577. https://doi.org/10.1021/jp972543+.
5. Gustafson, J.A.; Wilmer, C.E. Computational Design of Metal–Organic Framework Arrays for Gas Sensing: Influence of Array Size and Composition on Sensor Performance. *J. Phys. Chem. C* **2017**, *121*, 6033–6038. https://doi.org/10.1021/acs.jpcc.6b09740.
6. Gustafson, J.A.; Wilmer, C.E. Optimizing information content in MOF sensor arrays for analyzing methane-air mixtures. *Sens. Actuators B Chem.* **2018**, *267*, 483–493. https://doi.org/10.1016/j.snb.2018.04.049

7. Gustafson, J.A.; Wilmer, C.E. Intelligent Selection of Metal–Organic Framework Arrays for Methane Sensing via Genetic Algorithms. *ACS Sens.* **2019**, *4*, 1586–1593. https://doi.org/10.1021/acssensors.9b00268.
8. Burkardt, J. The Truncated Normal Distribution. 35.
9. MacKay, D.J.C.; Kay, D.J.C.M. *Information Theory, Inference and Learning Algorithms*; Cambridge University Press, 2003; ISBN 978-0-521-64298-9.
